# Liver–Metabolic Phenotypes and Renal Vulnerability in Community-Acquired Sepsis: Insights from the SepsisFAT Cohort

**DOI:** 10.3390/metabo16070468

**Published:** 2026-07-04

**Authors:** Lara Šamadan Marković, Hana Panić, Juraj Krznarić, Branimir Gjurašin, Neven Papić

**Affiliations:** 1 Croatian Institute of Public Health, 10000 Zagreb, Croatia; samadanlara@gmail.com; 2 Emergency Infectious Diseases Department, University Hospital for Infectious Diseases “Dr. Fran Mihaljević”, 10000 Zagreb, Croatia; hpanic@bfm.hr; 3 Department for Intensive Care, University Hospital for Infectious Diseases “Dr. Fran Mihaljević”, 10000 Zagreb, Croatia; jkrznaric@bfm.hr (J.K.); bgjurasin@bfm.hr (B.G.); 4 Department for Infectious Diseases, School of Medicine, University of Zagreb, 10000 Zagreb, Croatia; 5 Department for Viral Hepatitis, University Hospital for Infectious Diseases “Dr. Fran Mihaljević”, 10000 Zagreb, Croatia

**Keywords:** sepsis, MASLD, metabolic-dysfunction-associated steatotic liver disease, acute kidney injury, FIB-4, biochemical biomarkers, thromboinflammation, sepsis phenotypes, personalized medicine

## Abstract

**Highlights:**

**What are the main findings?**

- Liver–metabolic phenotyping identified a renal-vulnerable subgroup in community-acquired sepsis.
- The mixed liver–cardiometabolic phenotype had the highest AKI and CRRT risk, whereas cardiometabolic burden alone did not confer excess organ-support risk.

**What are the implications of the main findings?**

- MASLD-associated sepsis risk is heterogeneous and should not be interpreted as a simple binary exposure.
- Admission liver–metabolic phenotyping may support early renal surveillance and risk stratification beyond SOFA severity and baseline eGFR.

**Abstract:**

**Background:** Metabolic-dysfunction-associated steatotic liver disease (MASLD) is associated with adverse outcomes in sepsis, but risk stratification within MASLD remains insufficiently defined. We investigated whether an admission liver–metabolic phenotype framework combining cardiometabolic burden with liver injury/fibroinflammatory risk markers identifies clinically relevant organ-support vulnerability in community-acquired sepsis. **Methods**: This secondary analysis of the prospective SepsisFAT cohort (378 adults with community-acquired sepsis) classified patients into four phenotypes by cardiometabolic burden (≥2 of: diabetes, hypertension, dyslipidemia, BMI ≥ 30 kg/m^2^) and liver-risk positivity (FIB-4 ≥ 2.67, APRI ≥ 1.0, liver stiffness ≥ 10 kPa, or FAST ≥ 0.55). The primary outcome was acute kidney injury (AKI), while continuous renal replacement therapy (CRRT), other organ-support outcomes and in-hospital mortality were secondary endpoints. **Results**: Phenotype distribution was Low-risk 137 (36.2%), Cardiometabolic-only 84 (22.2%), Liver-dominant 88 (23.3%), and Mixed liver–cardiometabolic 69 (18.3%). AKI and CRRT increased across phenotypes (13.9% to 40.6% and 5.1% to 26.1%, respectively), and in-hospital mortality was highest in the Mixed phenotype (26.1%). After Firth-penalized adjustment for age, sex, and admission SOFA, the Mixed phenotype remained independently associated with AKI (aOR 2.82, 95% CI 1.37–5.90) and CRRT (aOR 3.87, 1.50–10.80), confirmed in non-renal SOFA and admission eGFR-adjusted sensitivity analyses. Cardiometabolic burden alone did not confer excess organ-support risk. The same gradient persisted within the MASLD subgroup. **Conclusions**: Admission liver–metabolic phenotyping identified a renal-vulnerable sepsis subgroup not captured by binary MASLD classification alone. These findings support prospective, multicenter external validation of liver–metabolic phenotyping as a pragmatic approach to renal risk stratification in community-acquired sepsis.

## 1. Introduction

Metabolic-dysfunction-associated steatotic liver disease (MASLD) is now the most prevalent chronic liver condition worldwide and is increasingly recognized as a systemic metabolic disorder that affects cardiovascular, renal, immune and microvascular systems [1,2,3,4,5]. These extra-hepatic manifestations are particularly relevant in acute infection, where coexisting metabolic and hepatic dysfunction may converge with sepsis-induced organ failure [6,7,8].

Several large population studies have linked MASLD with adverse infection-related outcomes, including severe infections requiring hospitalization and sepsis-related mortality [9,10,11,12]. In the prospective SepsisFAT cohort, MASLD was associated with higher in-hospital mortality, acute kidney injury (AKI), continuous renal replacement therapy (CRRT) and invasive mechanical ventilation (IMV) [13]. In a follow-up immunological analysis [14], MASLD patients displayed distinct cytokine kinetics during sepsis, providing biochemical evidence for biological diversity within MASLD. Whether this variety also manifests as a host-disease phenotype identifiable from early admission clinical, laboratory and elastography-based data has not been examined. Importantly, these analyses treated MASLD primarily as a binary diagnostic exposure, which may not capture the full spectrum of liver–metabolic vulnerability in acute infection.

MASLD itself is biologically heterogeneous, with emerging recognition of cardiometabolic-predominant, liver-specific and mixed subtypes that differ in clinical trajectory [1,15,16]. The cardiometabolic axis captures the systemic background of diabetes, hypertension, dyslipidemia and obesity, whereas the liver axis—measured non-invasively through FIB-4, APRI, FAST score and transient elastography (liver stiffness measurement, LSM) [1,17,18,19]—captures hepatocellular injury, fibrosis risk and fibroinflammatory activity. Whether these two aspects interact during sepsis to define a clinically interpretable risk stratification of organ-support outcomes has not, to our knowledge, been examined.

Therefore, we performed a secondary analysis of the SepsisFAT cohort using a predefined liver–metabolic phenotype framework. We hypothesized that combining cardiometabolic burden with liver injury/fibroinflammatory marker positivity at admission would identify a gradient of organ-support vulnerability—particularly acute kidney injury—beyond binary MASLD classification or acute sepsis-severity scoring alone. The framework was designed to rely on early admission clinical, laboratory and non-invasive liver assessment data.

## 2. Materials and Methods

### 2.1. Study Design and Population

This was a secondary analysis of the previously reported prospective SepsisFAT cohort (Clinical Study Identifier NCT06021743) [13], which enrolled adults hospitalized with community-acquired sepsis and evaluated MASLD as a binary exposure. The present analysis addressed a distinct question: whether an a priori liver–metabolic phenotype framework combining cardiometabolic burden and liver injury/fibroinflammatory risk markers identifies organ-failure and organ-support patterns beyond binary MASLD classification.

Adults hospitalized with community-acquired sepsis were eligible if sufficient clinical, laboratory and liver-related data were available to assign liver–metabolic phenotype and ascertain clinical outcomes. The final analytic cohort included 378 patients. Patients were treated according to standard institutional protocols for sepsis care. The study was approved by the Ethical Committee of the University Hospital for Infectious Diseases (UHID) in Zagreb, Croatia (protocol code 01-1247-2-2019 and approval granted on 30 August 2019), and informed consent procedures followed the original SepsisFAT protocol.

### 2.2. Clinical and Laboratory Data

Baseline demographic, clinical and laboratory variables were collected at hospital admission or within the early admission window defined in the original cohort protocol. Variables included age, sex, body mass index (BMI), cardiometabolic comorbidities, age-adjusted Charlson comorbidity index, admission SOFA score, infection source, inflammatory markers, renal function and liver-related parameters.

Cardiometabolic variables included type 2 diabetes, arterial hypertension, dyslipidemia and obesity, defined as BMI ≥ 30 kg/m^2^. Dyslipidemia was defined by documented medical history and/or current lipid-lowering therapy; lipid values were used when available but were not required because fasting lipid profiles were not systematically obtained during acute sepsis. Laboratory variables included aminotransferases, gamma-glutamyl transferase, platelet count, glucose, C-reactive protein, procalcitonin, D-dimer, leukocyte count, neutrophil-to-lymphocyte ratio, creatinine and estimated glomerular filtration rate. Transient elastography-derived variables included controlled attenuation parameter (CAP) and LSM. FAST score, FIB-4 and APRI were calculated using standard formulas.

### 2.3. Definition of MASLD

MASLD status was defined according to contemporary nomenclature requiring hepatic steatosis and cardiometabolic risk criteria. Hepatic steatosis was assessed primarily by transient elastography using FibroScan-derived CAP, as previously described [13]. In the present analysis, MASLD status was used for subgroup analyses, whereas the primary focus was the liver–metabolic phenotype framework.

### 2.4. Liver–Metabolic Phenotype Framework

The phenotype framework was designed to separate two biologically and clinically distinct but frequently overlapping dimensions of risk: cardiometabolic burden and liver injury/fibroinflammatory risk. Cardiometabolic burden was included to capture the systemic metabolic background commonly associated with MASLD, obesity, diabetes and cardiovascular risk. Liver-risk marker positivity was included to capture hepatic injury, fibroinflammatory activity or liver-related organ vulnerability using routinely available non-invasive markers. This two-axis approach was chosen to test whether adverse sepsis outcomes were associated with cardiometabolic burden alone, liver-risk marker positivity alone, or their coexistence.

Patients were categorized into four mutually exclusive liver–metabolic phenotypes using these two axes. Cardiometabolic burden was defined as the presence of at least two of the following: type 2 diabetes, arterial hypertension, dyslipidemia or BMI ≥ 30 kg/m^2^. This threshold was selected to capture broader metabolic burden rather than isolated risk factors. Liver-risk marker positivity was defined as the presence of at least one of the following: FIB-4 ≥ 2.67 [18], APRI ≥ 1.0 [19], LSM ≥ 10 kPa [1] or FAST score ≥ 0.55 [17]. These established thresholds were applied without modification to preserve comparability with prior liver-risk literature and clinical practice. In this acute sepsis cohort, however, liver-risk positivity was interpreted as a composite clinical profile rather than as definitive chronic fibrosis staging, because aminotransferases, platelet count and liver stiffness may be influenced by acute systemic illness, including inflammation, thrombocytopenia, hemodynamic instability or transient liver injury.

### 2.5. Outcomes

The primary outcome was acute kidney injury, defined according to KDIGO criteria [20]. Secondary outcomes included continuous renal replacement therapy (CRRT), septic shock, ICU admission, IMV, acute respiratory distress syndrome (ARDS) and in-hospital mortality, which were defined according to the original SepsisFAT protocol and standard definitions.

Two composite outcomes were defined: the renal–circulatory composite, comprising AKI, CRRT or septic shock; and the organ-support composite, comprising ICU admission, IMV, CRRT or septic shock. Mortality was analyzed as in-hospital mortality and was considered an exploratory secondary outcome.

### 2.6. Statistical Analysis

Continuous variables are presented as median with interquartile range and categorical variables as counts and percentages. Between-group comparisons were performed using the Kruskal–Wallis test for continuous variables and the χ^2^ or Fisher’s exact test for categorical variables, as appropriate. Trends across ordered phenotypes were assessed using the Cochran–Armitage trend test. Crude odds ratios were calculated using the Low-risk phenotype as the reference category.

Severity-adjusted associations between phenotype and organ-support outcomes were assessed using Firth-penalized logistic regression adjusted for age, sex and admission SOFA score. Because SOFA includes renal impairment, sensitivity analyses for AKI and CRRT used non-renal SOFA, calculated as total SOFA minus the admission creatinine-based renal SOFA component. To further address the potential influence of reduced renal function at presentation, an additional sensitivity analysis for AKI and CRRT was performed using Firth-penalized logistic regression adjusted for age, sex, non-renal SOFA score, and admission eGFR. This was specifically designed to evaluate whether the phenotype association with renal outcomes persisted beyond differences in baseline renal function across groups.

In-hospital mortality was analyzed using sequential logistic regression models: unadjusted, adjusted for age and sex, and adjusted for age, sex and respiratory versus non-respiratory infection source (pre-specified primary mortality model). Because admission SOFA score reflects acute organ dysfunction that may lie on the causal pathway between phenotype and mortality, additional adjustment for SOFA was performed as a pre-specified sensitivity analysis. Sensitivity mortality models used age-adjusted Charlson comorbidity index instead of age to avoid double adjustment for age. Sensitivity analyses repeated the phenotype framework using each liver-risk marker individually to assess robustness to marker choice. Additional exploratory analyses included within-MASLD subgroup analyses and two-axis models including cardiometabolic burden, liver-risk positivity and their interaction. Missing values were not imputed; regression analyses were complete-case analyses. Multicollinearity was assessed using variance inflation factors; all VIFs in the severity-adjusted models were < 1.5, indicating no concerning collinearity between covariates. All tests were two-sided, and *p* < 0.05 was considered statistically significant.

For the pre-specified renal endpoints (AKI and CRRT), *p*-values for the three phenotype contrasts versus Low risk were additionally adjusted for multiple comparisons using the Benjamini–Hochberg false discovery rate procedure, with separate correction within the crude and severity-adjusted analysis layers. All other organ-support outcomes (septic shock, ICU admission, IMV, ARDS, composite endpoints) and in-hospital mortality were considered exploratory secondary outcomes and were not included in the primary FDR analysis.

Statistical analyses were performed using R version 4.6.0 (R Foundation for Statistical Computing, Vienna, Austria).

## 3. Results

### 3.1. Cohort Characteristics and Phenotype Distribution

**Figure 1:**
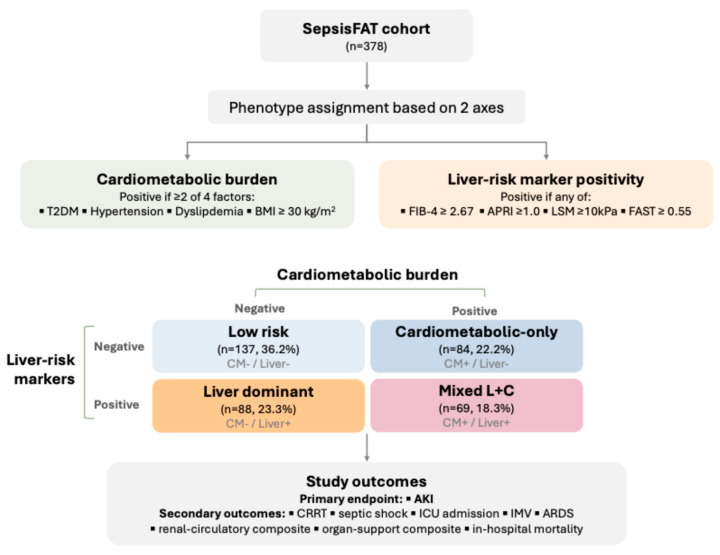
Study design and liver–metabolic phenotype classification. APRI, AST-to-platelet ratio index; BMI, body mass index; CM, cardiometabolic; FAST, FibroScan-AST score; FIB-4, Fibrosis-4 index; L + C, liver–cardiometabolic; LSM, liver stiffness measurement.

Of 378 adults hospitalized with community-acquired sepsis, 174 (46.0%) fulfilled criteria for MASLD. Using the liver–metabolic phenotype framework, 137 patients (36.2%) were classified as Low risk, 84 (22.2%) as Cardiometabolic-only, 88 (23.3%) as Liver-dominant and 69 (18.3%) as Mixed liver–cardiometabolic. Overall, 157 patients (41.5%) had at least one elevated liver injury/fibroinflammatory risk marker, and 153 patients (40.5%) fulfilled criteria for cardiometabolic burden (Figure 1, Supplementary Figure S1).

Baseline characteristics differed across phenotypes (Table 1). Cardiometabolic-only and Mixed liver–cardiometabolic patients had higher BMI, glucose levels and a higher prevalence of type 2 diabetes, arterial hypertension, dyslipidemia and obesity. Liver-dominant and Mixed liver–cardiometabolic patients had higher liver-related risk markers, including LSM, FIB-4, APRI, FAST score and aminotransferases. The Mixed liver–cardiometabolic phenotype also had the highest age-adjusted Charlson comorbidity index, admission SOFA score and creatinine, and the lowest admission eGFR.

Inflammatory profiles differed between phenotypes in patterns consistent with the framework’s underlying axes. Procalcitonin and D-dimer were higher in liver-risk positive phenotypes, whereas CRP and neutrophil-to-lymphocyte ratio did not differ significantly across groups. Platelet count was substantially lower in liver-risk-positive phenotypes (Liver-dominant 138 × 10^9^/L; Mixed L + C 180 × 10^9^/L) compared with Cardiometabolic-only (252 × 10^9^/L) and Low-risk (246 × 10^9^/L) groups (*p* < 0.001). Consequently, the systemic immune–inflammation index was reduced in liver-risk groups, primarily reflecting lower platelet counts rather than a blunted inflammatory response. Because thrombocytopenia in sepsis is multifactorial and may reflect consumption, DIC, hepatic sequestration, marrow suppression or overall disease severity, the lower platelet counts observed in the Liver-dominant and Mixed liver–cardiometabolic phenotypes should be interpreted cautiously.

Infection source distribution and bacteremia rates did not differ significantly between phenotypes (overall infection-source distribution *p* = 0.261; respiratory vs. non-respiratory source *p* = 0.192; bacteremia *p* = 0.163) (Supplementary Table S1).

### 3.2. AKI Incidence Across Liver–Metabolic Phenotypes

**Figure 2:**
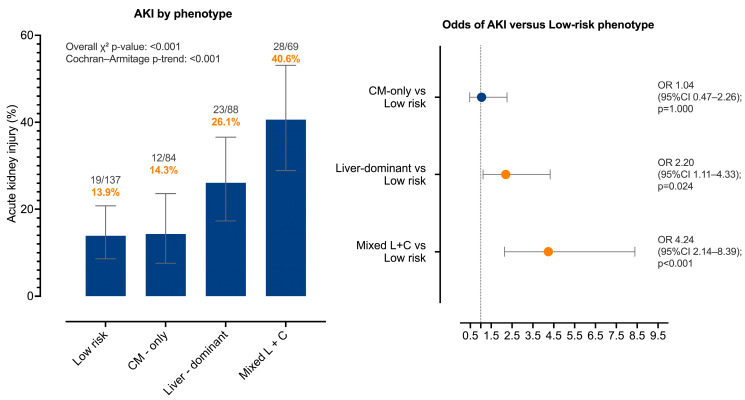
Acute kidney injury by liver–metabolic phenotype. Bar chart showing AKI incidence across the four liver–metabolic phenotypes, with error bars representing 95% binomial confidence intervals. AKI incidence increased stepwise from Low-risk and Cardiometabolic-only phenotypes to Liver-dominant and Mixed liver–cardiometabolic phenotypes (overall χ^2^ *p* < 0.001; Cochran–Armitage *p*-trend < 0.001). Inset forest plot shows odds ratios for AKI versus the Low-risk phenotype. Orange circles highlight the liver-involved phenotypes. AKI, acute kidney injury; CI, confidence interval; OR, odds ratio.

AKI incidence differed significantly across liver–metabolic phenotypes and showed a stepwise increase in liver-risk positive groups (Figure 2). AKI occurred in 13.9% of Low-risk patients, 14.3% of Cardiometabolic-only patients, 26.1% of Liver-dominant patients and 40.6% of Mixed liver–cardiometabolic patients (overall χ^2^ *p* < 0.001; *p*-trend < 0.001). Compared with the Low-risk phenotype, the odds of AKI were not increased in the Cardiometabolic-only phenotype (OR 1.04, 95% CI 0.47–2.26, *p* = 1.000) but were higher in the Liver-dominant phenotype (OR 2.20, 95% CI 1.11–4.33, *p* = 0.024) and highest in the Mixed liver–cardiometabolic phenotype (OR 4.24, 95% CI 2.14–8.39, *p* < 0.001). These findings suggest that higher AKI rates were observed primarily in liver-risk-positive phenotypes, particularly when combined with cardiometabolic burden, whereas cardiometabolic burden alone was not associated with increased AKI risk.

These contrasts remained statistically significant after Benjamini–Hochberg adjustment for multiple comparisons across the six primary/key-secondary tests (Mixed L + C vs. Low: q < 0.001 crude, q = 0.020 severity-adjusted; Liver-dominant vs. Low: q = 0.035 crude; Supplementary Table S12).

### 3.3. Organ-Support Outcomes Across Liver–Metabolic Phenotypes

**Figure 3:**
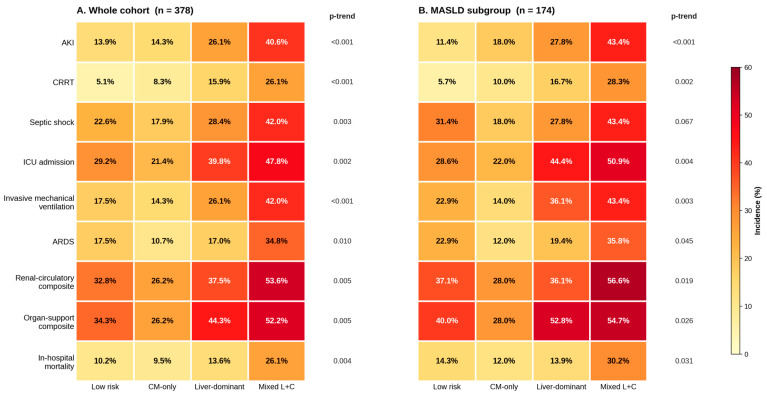
Organ-support outcomes by liver–metabolic phenotype in the whole cohort and within the MASLD subgroup. Heatmaps showing the incidence of renal, respiratory, circulatory, composite organ-support outcomes and in-hospital mortality across liver–metabolic phenotypes in the overall SepsisFAT cohort (**A**) and among patients with MASLD (**B**). Values represent event incidence (%). *p*-trend was calculated using the Cochran–Armitage trend test across ordered phenotypes. AKI, acute kidney injury; ARDS, acute respiratory distress syndrome; CRRT, continuous renal replacement therapy; ICU, intensive care unit; L + C, liver–cardiometabolic; MASLD, metabolic-dysfunction-associated steatotic liver disease.

The liver–metabolic phenotype framework showed consistent separation across crude organ-support outcomes (Table 2; Figure 3, full results with overall χ^2^ *p*-values in Supplementary Table S2). CRRT occurred in 5.1% Low-risk patients, 8.3% Cardiometabolic-only patients, 15.9% Liver-dominant patients and 26.1% Mixed liver–cardiometabolic patients (overall χ^2^ *p* < 0.001; *p*-trend < 0.001). Compared with Low-risk patients, the Mixed liver–cardiometabolic phenotype had markedly higher odds of CRRT (OR 6.55, 95% CI 2.58–16.63). Following Benjamini–Hochberg correction across the six primary/key-secondary tests, the CRRT association remained significant for both Mixed L + C vs. Low (q < 0.001 crude, q = 0.020 severity-adjusted) and Liver-dominant vs. Low (q = 0.020 crude; Supplementary Table S12).

A similar pattern was observed for non-renal organ-support outcomes. The occurrence of septic shock increased from 22.6% in the Low-risk phenotype and 17.9% in the Cardiometabolic-only phenotype to 28.4% in the Liver-dominant phenotype and 42.0% in the Mixed liver–cardiometabolic phenotype (*p* = 0.005; *p*-trend = 0.003). ICU admission rates were 29.2%, 21.4%, 39.8% and 47.8%, respectively (*p* = 0.002; *p*-trend = 0.002), while invasive mechanical ventilation (IMV) was required in 17.5%, 14.3%, 26.1% and 42.0% (*p* < 0.001; *p*-trend < 0.001). ARDS was also most frequent in the Mixed phenotype (34.8%; *p* = 0.002; *p*-trend = 0.010) (Table 2, Figure 3).

Composite endpoints showed the same pattern. The renal–circulatory composite rates were 32.8% of Low-risk, 26.2% of Cardiometabolic-only, 37.5% of Liver-dominant and 53.6% of Mixed liver–cardiometabolic patients (*p* = 0.004; *p*-trend = 0.005). The organ-support composite reached 34.3%, 26.2%, 44.3% and 52.2%, respectively (*p* = 0.004; *p*-trend = 0.005).

### 3.4. Cardiometabolic Burden Alone Is Not Associated with Excess Organ-Support Risk

A key observation was that cardiometabolic burden alone did not identify a high-risk sepsis subgroup. Despite higher BMI, glucose levels and a substantially higher prevalence of type 2 diabetes, arterial hypertension, dyslipidemia and obesity, Cardiometabolic-only patients had outcome rates comparable to, or numerically lower than, Low-risk patients across several endpoints. AKI was nearly identical between Cardiometabolic-only and Low-risk patients (14.3% vs. 13.9%), and Cardiometabolic-only patients had lower rates of septic shock, ICU admission, IMV, ARDS, renal–circulatory composite and organ-support composite.

In contrast, outcome rates increased in phenotypes characterized by liver-risk marker positivity. Liver-dominant patients had higher rates of AKI, CRRT, ICU admission and composite organ-support outcomes than Cardiometabolic-only patients, while the Mixed liver–cardiometabolic phenotype had the highest event rates across all outcomes. These findings suggest that excess organ-support vulnerability was not explained by cardiometabolic burden alone but was concentrated in patients with liver injury/fibroinflammatory risk marker positivity, particularly when this coexisted with cardiometabolic burden.

In exploratory two-axis logistic models including cardiometabolic burden, liver-risk positivity and their interaction (Supplementary Table S3), cardiometabolic burden alone was not associated with AKI or CRRT. By contrast, liver-risk positivity was associated with AKI (OR 2.31, 95% CI 1.13–4.75, *p* = 0.022) and CRRT (OR 3.48, 95% CI 1.38–9.55, *p* = 0.010). Interaction terms were not significant for AKI or CRRT, suggesting that the renal signal was driven primarily by the liver-risk axis, while the highest absolute event rates occurred when liver-risk positivity coexisted with cardiometabolic burden. An exploratory significant interaction was observed for ARDS (OR 3.54, 95% CI 1.16–11.38, *p* = 0.030), suggesting a possible interaction signal for respiratory outcomes; this exploratory finding requires prospective replication.

### 3.5. Severity-Adjusted Associations with AKI and CRRT

**Figure 4:**
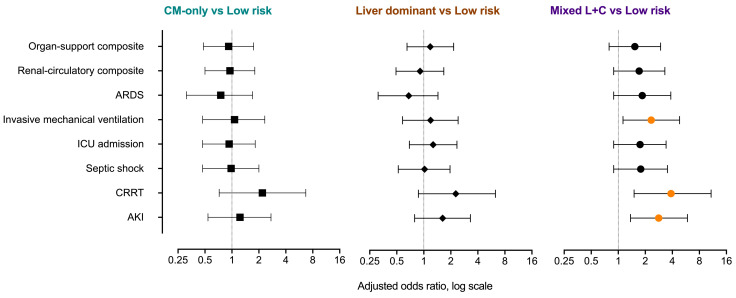
Severity-adjusted associations between liver–metabolic phenotypes and organ-support outcomes. Forest plot showing adjusted odds ratios and 95% confidence intervals from Firth-penalized logistic regression models adjusted for age, sex and admission SOFA score. The Low-risk phenotype was used as the reference category. The *x*-axis is displayed on a logarithmic scale. Orange symbols indicate statistically significant associations after adjustment. AKI, acute kidney injury; ARDS, acute respiratory distress syndrome; CRRT, continuous renal replacement therapy; ICU, intensive care unit; L + C, liver–cardiometabolic.

Firth-penalized logistic regression models adjusted for age, sex and admission SOFA score were used to assess whether phenotype–outcome associations persisted beyond baseline sepsis severity. Complete-case severity-adjusted models included 371 patients (Table 3; Figure 4; Supplementary Table S4).

After adjustment, the Mixed liver–cardiometabolic phenotype remained independently associated with the primary endpoint AKI (aOR 2.82, 95% CI 1.37–5.90, *p* = 0.005) and with CRRT (aOR 3.87, 95% CI 1.50–10.80, *p* = 0.005), compared with the Low-risk phenotype. The Mixed phenotype was also associated with IMV (aOR 2.33, 95% CI 1.13–4.82, *p* = 0.023). Associations with septic shock, ICU admission, ARDS and composite organ-support outcomes were attenuated after SOFA adjustment and were no longer statistically significant.

Admission SOFA score was consistently associated with all organ-support outcomes, whereas Cardiometabolic-only and Liver-dominant phenotypes were not independently associated with outcomes after severity adjustment. These findings indicate that the Mixed liver–cardiometabolic phenotype retained its strongest incremental prognostic signal for renal outcomes, particularly AKI and need for CRRT, beyond global sepsis severity at presentation.

Because total SOFA includes renal dysfunction, sensitivity models for AKI and CRRT were repeated using creatinine-based non-renal SOFA instead of total SOFA. In these models, the Mixed liver–cardiometabolic phenotype remained independently associated with AKI (aOR 3.16, 95% CI 1.55–6.55, *p* = 0.002) and CRRT (aOR 4.44, 95% CI 1.74–12.22, *p* = 0.002), supporting that the renal signal was not solely driven by the renal component of baseline SOFA (Supplementary Table S5).

Because the Mixed liver–cardiometabolic phenotype had substantially lower median admission eGFR (47.4 vs. 83.6 mL/min/1.73 m^2^ in Low risk, with 52.9% versus 25.2% of patients having eGFR < 60), an additional sensitivity analysis was performed including both non-renal SOFA and admission eGFR as covariates (*n* = 369). In this model, lower admission eGFR was independently associated with AKI (aOR 0.99 per mL/min/1.73 m^2^, 95% CI 0.98–1.00, *p* = 0.036) and CRRT (aOR 0.99, 95% CI 0.98–1.00, *p* = 0.049). Importantly, the Mixed liver–cardiometabolic phenotype remained independently associated with both AKI (aOR 2.72, 95% CI 1.31–5.71, *p* = 0.007) and CRRT (aOR 3.73, 95% CI 1.43–10.43, *p* = 0.007), indicating that the renal signal was not solely explained by lower baseline renal function in this subgroup (Supplementary Table S6). In an additional sensitivity analysis excluding patients with documented pre-existing chronic kidney disease (*n* = 22), the Mixed liver–cardiometabolic phenotype estimates remained essentially unchanged for both AKI (aOR 2.77, 95% CI 1.29–5.95, *p* = 0.009; *n* = 349) and CRRT (aOR 3.41, 95% CI 1.23–9.44, *p* = 0.018), confirming that the renal signal was not driven by pre-existing chronic kidney disease.

Sensitivity analyses using individual liver-risk markers supported the robustness of the renal signal, particularly for bloodwork-based and FAST-based definitions. When liver-risk positivity was defined separately by FIB-4, APRI or FAST, the mixed liver–cardiometabolic phenotype remained associated with AKI and CRRT, whereas LSM alone did not reproduce the renal association (Supplementary Table S7).

### 3.6. Phenotype Patterns Within the MASLD Subgroup

Within the MASLD subgroup, organ-support outcomes varied substantially across liver–metabolic profiles (Figure 3B). Among 174 patients with MASLD, 35 were classified as Low-risk MASLD, 50 as Cardiometabolic-only MASLD, 36 as Liver-dominant/fibroinflammatory MASLD and 53 as Mixed liver–cardiometabolic MASLD.

The strongest separation was observed for renal outcomes (Figure 3B; Supplementary Table S8). AKI increased from 11.4% in Low-risk MASLD and 18.0% in Cardiometabolic-only MASLD to 27.8% in Liver-dominant/fibroinflammatory MASLD and 43.4% in Mixed liver–cardiometabolic MASLD (overall χ^2^ *p* = 0.003; *p*-trend < 0.001). Compared with Low-risk MASLD, the Mixed liver–cardiometabolic MASLD profile was associated with higher odds of AKI (OR 5.94, 95% CI 1.84–19.23). CRRT followed a similar gradient, occurring in 5.7%, 10.0%, 16.7% and 28.3% of patients, respectively (*p* = 0.018; *p*-trend = 0.002; OR for Mixed vs. Low-risk MASLD 6.51, 95% CI 1.39–30.61).

Other organ-support outcomes showed the same directional pattern. The Mixed liver–cardiometabolic MASLD profile had the highest rates of ICU admission, IMV, ARDS and composite organ-support outcomes, whereas the Cardiometabolic-only MASLD profile generally did not show excess risk despite the presence of cardiometabolic burden. In exploratory severity-adjusted Firth models within the MASLD subgroup, the Mixed liver–cardiometabolic MASLD profile remained associated with AKI compared with Low-risk MASLD (aOR 4.24, 95% CI 1.22–17.39, *p* = 0.022), while associations with other outcomes were directionally consistent but no longer statistically significant (Supplementary Table S9).

Together, these findings indicate that MASLD-associated sepsis vulnerability was heterogeneous and concentrated in patients with combined cardiometabolic burden and liver-risk marker positivity, rather than uniformly distributed across MASLD as a diagnostic category.

### 3.7. Mortality Across Phenotypes and Sequential Adjustment Models

**Figure 5:**
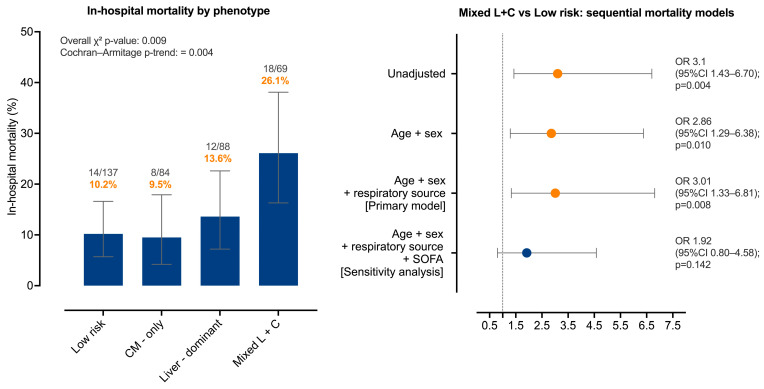
In-hospital mortality across liver–metabolic phenotypes and sequential adjustment models. Crude in-hospital mortality across the four liver–metabolic phenotypes, with error bars representing exact binomial 95% confidence intervals is shown. Overall, between-group comparison was performed using the χ^2^ test, and trend across ordered phenotypes was assessed using the Cochran–Armitage trend test. Odds ratios for in-hospital mortality comparing the Mixed liver–cardiometabolic phenotype with the Low-risk phenotype: the pre-specified primary model (age + sex + respiratory source) and a sensitivity model with additional adjustment for admission SOFA, which may lie on the causal pathway between phenotype and mortality are shown on the right. Orange symbols indicate statistically significant associations after adjustment aOR, adjusted odds ratio; CI, confidence interval; L + C, liver–cardiometabolic; OR, odds ratio; SOFA, Sequential Organ Failure Assessment.

In-hospital mortality differed across liver–metabolic phenotypes and was highest in the Mixed liver–cardiometabolic group. Mortality was 10.2% in the Low-risk phenotype, 9.5% in the Cardiometabolic-only phenotype, 13.6% in the Liver-dominant phenotype and 26.1% in the Mixed liver–cardiometabolic phenotype (overall χ^2^ *p* = 0.009; *p*-trend = 0.004) (Figure 5). Compared with Low-risk patients, the Mixed liver–cardiometabolic phenotype had higher unadjusted odds of in-hospital death (OR 3.10, 95% CI 1.43–6.70, *p* = 0.004).

Within the MASLD subgroup, mortality followed a similar pattern and was highest among patients with the mixed liver–cardiometabolic MASLD profile. Mortality was 14.3% in Low-risk MASLD, 12.0% in Cardiometabolic-only MASLD, 13.9% in Liver-dominant/fibroinflammatory MASLD and 30.2% in Mixed liver–cardiometabolic MASLD. The trend across MASLD phenotypes was significant (*p*-trend = 0.031), although the overall between-group comparison was borderline (*p* = 0.067).

Because respiratory infection was associated with higher mortality in the cohort, the primary mortality model was adjusted for age, sex and respiratory infection source. In this primary model, the Mixed liver–cardiometabolic phenotype remained independently associated with in-hospital mortality (aOR 3.01, 95% CI 1.33–6.81, *p* = 0.008). Because admission SOFA score reflects acute organ dysfunction that may itself be partly mediated by the phenotype (i.e., the Mixed liver–cardiometabolic phenotype may exert its mortality effect in part through a higher admission SOFA), additional adjustment for SOFA was performed as a pre-specified sensitivity analysis rather than as the primary model. In this sensitivity analysis the association was attenuated and no longer statistically significant (aOR 1.92, 95% CI 0.80–4.58, *p* = 0.142), a pattern consistent with partial mediation of the phenotype–mortality association by overall illness severity at admission (Figure 5; Supplementary Table S10). Sensitivity models using age-adjusted Charlson comorbidity index instead of age yielded similar results: the association remained significant after adjustment for sex, age-adjusted Charlson index and respiratory source (aOR 2.99, 95% CI 1.33–6.72, *p* = 0.008) but was attenuated after additional SOFA adjustment (aOR 2.04, 95% CI 0.86–4.82, *p* = 0.104) (Supplementary Table S11).

## 4. Discussion

In this secondary analysis of the SepsisFAT cohort, an a priori liver–metabolic phenotype framework was associated with variation in organ-support outcomes in adults hospitalized with community-acquired sepsis. Several patterns emerged from this analysis. First, the Mixed liver–cardiometabolic phenotype had the highest rates of AKI, CRRT, septic shock, ICU admission, IMV, ARDS and composite organ-support outcomes. Second, this phenotype remained independently associated with AKI and CRRT after adjustment for age, sex and admission SOFA, including sensitivity analysis using non-renal SOFA. Third, cardiometabolic burden alone—despite high MASLD prevalence among these patients—did not identify a high-risk subgroup; excess renal risk emerged only when it coexisted with liver-risk marker positivity. Fourth, within MASLD, adverse outcomes were concentrated in patients with the mixed profile rather than uniformly distributed across MASLD as a diagnostic category.

These results extend the original SepsisFAT report, which evaluated MASLD as a binary exposure [13], by shifting the focus from steatosis alone to the liver–metabolic architecture of risk in sepsis. The present phenotype framework refines, rather than contradicts, our prior finding that MASLD is associated with worse sepsis outcomes [13]: although the binary MASLD versus non-MASLD comparison reproduced the original signal of higher AKI, CRRT and mortality, excess renal risk was localized disproportionately to the Mixed liver–cardiometabolic phenotype. This pattern was also evident within the MASLD subgroup itself. Rather than indicating uniform risk across all MASLD patients, the within-MASLD analysis showed the steepest gradients for AKI (11.4% in low-risk MASLD to 43.4% in mixed MASLD) and CRRT (5.7% to 28.3%) in patients with combined cardiometabolic burden and liver-risk marker positivity; this mixed profile remained independently associated with AKI after severity adjustment (aOR 4.24, 95% CI 1.22–17.39). By contrast, the Cardiometabolic-only MASLD profile, despite substantial metabolic burden, showed a less consistent organ-support signal. However, the absence of a clear organ-support signal in the Cardiometabolic-only phenotype should be interpreted cautiously, as it may reflect true biology but could also be influenced by subgroup size, the operational definition of cardiometabolic burden, unmeasured treatment differences and limited power for secondary endpoints. These findings support viewing MASLD in sepsis as a heterogeneous host phenotype rather than as a binary exposure, with renal vulnerability appearing greatest when cardiometabolic dysfunction coexists with liver-risk marker positivity.

Our approach differs from the dominant data-driven sepsis phenotyping literature (Seymour et al. [21], α/β/γ/δ phenotypes; Calfee et al. [22], ARDS phenotypes; Davenport et al. [23], sepsis response signatures (SRS) endotypes) in being predefined, smaller in scale, and focused specifically on within-MASLD heterogeneity. As such, our framework is not a competitor to those large-scale clustering efforts, but rather a clinically oriented complement that may help interpret liver–metabolic comorbidity patterns within future precision-sepsis research.

Associations with renal outcomes were the most consistent finding across analyses. AKI and CRRT increased stepwise across phenotypes, persisted after age, sex and SOFA adjustment, and remained robust in non-renal SOFA sensitivity models, indicating that the framework was associated with renal susceptibility not fully explained by global sepsis severity or by the renal SOFA component. Mortality followed a parallel but less independent pattern: in-hospital mortality was highest in the Mixed phenotype, with an age–sex–respiratory-adjusted odds ratio of 3.01. Because admission SOFA captures acute organ dysfunction that may itself be partly driven by the liver–metabolic phenotype, the SOFA-adjusted estimate is best interpreted as a conservative lower bound on the phenotype–mortality association rather than as the primary effect estimate. This is consistent with at least partial mediation of the phenotype–mortality effect through overall illness severity at admission.

A biological explanation for this renal-predominant signal is consistent with the emerging concept of a hepatorenal–immunometabolic component in steatotic liver disease. Outside sepsis, MASLD is independently associated with chronic kidney disease and AKI through endothelial dysfunction, gut–liver–kidney inflammatory signaling, altered xenobiotic handling and impaired microcirculation [24,25,26,27,28]. In sepsis, the liver participates centrally in bacterial clearance, acute-phase synthesis, coagulation and metabolic homeostasis, and hepatic injury arises through hypoxic, cholestatic, inflammatory and mitochondrial mechanisms [8,29].

In the acute sepsis setting, FIB-4, APRI, FAST and LSM should be interpreted as composite biomarkers reflecting several overlapping biological processes rather than chronic fibrosis alone. Aminotransferase elevation may reflect hepatocellular stress from hypoxic, cholestatic, inflammatory and mitochondrial injury [8,29]. In addition, the shared dependence of FIB-4 and APRI on platelet count means that sepsis-associated thrombocytopenia may directly increase these scores. Because thrombocytopenia in sepsis is multifactorial and may reflect consumption, endothelial activation, disseminated thromboinflammation, DIC, hepatic sequestration or overall disease severity, the platelet-axis interpretation offered here should be viewed as one plausible contributor rather than as the exclusive mechanism [30,31,32].

Liver stiffness in acute illness may similarly be influenced by hepatic congestion, microcirculatory dysfunction and inflammatory edema, independent of chronic fibrosis [33,34]. Together, these markers may serve as integrated indicators of hepatocellular stress, platelet-axis disturbance, endothelial–microcirculatory injury and impaired hepatic reserve. Marker-specific sensitivity analyses supported this interpretation but also showed that not all components of the liver-risk definition contributed equally to the renal signal. FIB-4-, APRI- and FAST-defined liver-risk positivity reproduced the association of the Mixed liver–cardiometabolic phenotype with AKI and CRRT, whereas LSM alone did not. This suggests that the outcome-relevant component of hepatic vulnerability in acute sepsis may be driven more by hepatocellular injury, platelet-axis disturbance and FAST-captured steatotic/fibroinflammatory activity than by mechanical liver stiffness alone.

**Figure 6:**
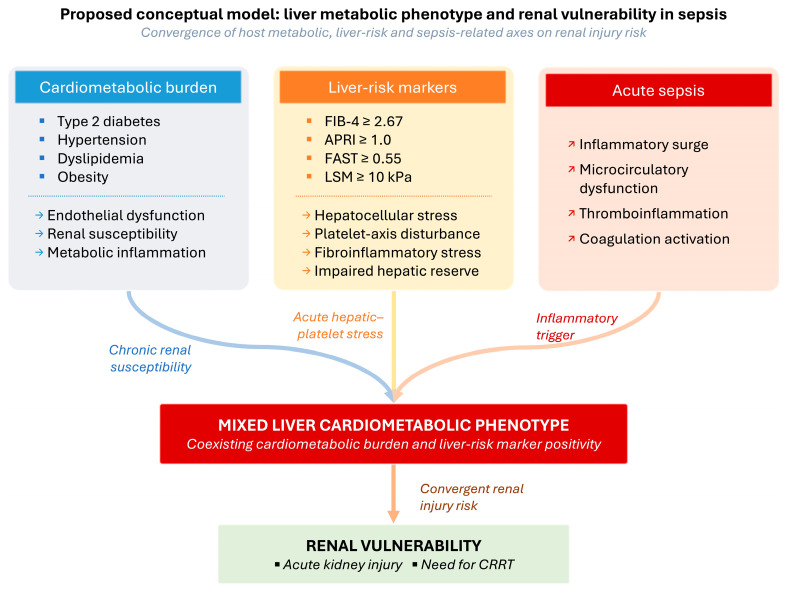
Proposed conceptual model linking liver–metabolic phenotype to renal vulnerability in community-acquired sepsis. Cardiometabolic burden, liver-risk marker positivity and acute sepsis-related stress may converge in the Mixed liver–cardiometabolic phenotype, extending beyond binary MASLD classification. This convergence may increase renal injury risk, clinically reflected by acute kidney injury and need for continuous renal replacement therapy. The schematic is hypothesis-generating and requires validation in mechanistic studies. L + C, liver–cardiometabolic.

Figure 6 provides a conceptual framework for interpreting the observed relationship between liver-risk marker positivity, cardiometabolic burden and adverse renal outcomes, possibly through hepatorenal–thromboinflammatory mechanisms previously described in steatotic liver disease and severe sepsis [28,35,36]. Recent observations linking elevated admission FIB-4 with sepsis mortality and renal replacement need further support this interpretation [37,38,39,40]. In our cohort, liver-risk-positive phenotypes had lower platelet counts and higher D-dimer and procalcitonin levels, whereas CRP, neutrophil-to-lymphocyte ratio and infection-site distribution did not differ significantly across groups. These findings suggest that the renal signal may reflect a hepatic–platelet–endothelial vulnerability phenotype rather than non-specific systemic inflammation or infection source alone.

Clinically, the framework is attractive because it uses early admission clinical, laboratory and non-invasive liver assessment data. If externally validated, it may provide a pragmatic basis for closer renal surveillance, nephrotoxin avoidance and cautious hemodynamic management in patients with the Mixed phenotype. These findings support further validation of liver–metabolic phenotyping as a pragmatic, admission-based approach to risk stratification in sepsis. Integration of this clinical–biochemical phenotype with proteomic, metabolomic, and gene expression layers (as exemplified by sepsis and ARDS endotypes [21,22,23]) is a next step toward better-resolved precision approaches in sepsis.

Several limitations should be considered when interpreting these findings. This is a single-center, observational, post hoc secondary analysis of a previously published cohort. The four-phenotype framework was specified a priori but was not pre-registered, and findings have not been externally validated. Results should therefore be interpreted as hypothesis-generating rather than as a validated risk stratification tool. The use of liver-risk markers (FIB-4, APRI, FAST, LSM) in the acute sepsis setting reflects acute as well as chronic liver state, and the interpretation of these markers in this context is exploratory; we therefore interpret these markers as liver injury/fibroinflammatory rather than definitive fibrosis measures. SOFA adjustment may partly over-adjust for early organ dysfunction, particularly for renal outcomes, although non-renal SOFA sensitivity analyses supported the AKI and CRRT findings; if some patients had AKI at or near admission, the phenotype should be viewed as identifying early renal vulnerability rather than strictly predicting incident AKI. Notably, admission eGFR was lower in the Mixed liver–cardiometabolic phenotype, reflecting a higher prevalence of reduced renal function at presentation; however, the phenotype–renal outcome associations persisted in sensitivity analyses additionally adjusted for admission eGFR, supporting that the renal signal is not solely attributable to early renal dysfunction. Because age contributes to the FIB-4 formula and was also included as a covariate, we verified that this did not introduce concerning collinearity (all VIFs < 1.5) and that the phenotype estimates were essentially unchanged in sensitivity models omitting age. Admission lactate was incompletely recorded (60.1%) and fluid balance was not systematically collected; therefore, neither could be incorporated as covariates, although median lactate values were broadly similar across phenotypes. Mortality and interaction subgroup analyses were limited by event numbers, and the absence of direct mechanistic measurements (metabolomics, endothelial markers, immune–metabolic assays) means biological pathways remain inferential.

In conclusion, an a priori liver–metabolic phenotype framework identified a renal-vulnerable subgroup among adults with community-acquired sepsis, defined by the coexistence of cardiometabolic burden and liver injury/fibroinflammatory marker positivity. Within MASLD, the same framework highlighted clinically relevant heterogeneity in organ-support risk. These hypothesis-generating findings describe a clinical phenotype-based approach to within-MASLD risk heterogeneity in sepsis that warrants external validation. If confirmed, integration with molecular characterization (transcriptomic, proteomic, metabolomic) could further refine sepsis precision medicine.

## 5. Conclusions

In community-acquired sepsis, a pre-specified liver–metabolic framework identified a clinically meaningful subgroup at increased risk of AKI, CRRT, and other organ-support outcomes. Cardiometabolic burden alone was insufficient to define this vulnerable phenotype; the strongest prognostic signal was concentrated in patients with concurrent liver-risk marker positivity, including within MASLD. These exploratory findings support early bedside risk stratification that integrates cardiometabolic and liver-related features beyond binary MASLD classification.

## Figures and Tables

**Table 1:** Baseline characteristics by liver-metabolic phenotype (SepsisFAT cohort, *n* = 378).

	Low Risk (*n* = 137)	Cardiometabolic-Only (*n* = 84)	Liver-Dominant (*n* = 88)	Mixed L + C (*n* = 69)	*p*-Value
Demographics and sepsis severity					
Age, years	62.7 (49.2–75.5)	70.0 (62.8–79.0)	64.1 (47.4–75.2)	71.2 (62.4–79.1)	<0.001
Male sex	66 (48.2)	35 (41.7)	49 (55.7)	37 (53.6)	0.264
SOFA score	2.0 (1.5–4.0)	2.0 (1.0–3.0)	3.0 (2.0–6.0)	4.0 (2.0–6.0)	<0.001
BMI, kg/m^2^	24.7 (22.6–27.1)	30.1 (25.0–33.1)	26.1 (23.9–28.8)	30.7 (28.1–35.6)	<0.001
Respiratory infection source	49 (35.8)	19 (22.6)	26 (29.5)	24 (34.8)	0.192
Comorbidities and phenotype-defining components					
Age-adjusted CCI	3.0 (1.0–5.0)	4.0 (3.0–6.0)	2.5 (1.0–4.0)	4.5 (3.0–6.0)	<0.001
CKD	3 (2.2)	7 (8.3)	4 (4.5)	8 (11.6)	0.012
MASLD	35 (25.5)	50 (59.5)	36 (40.9)	53 (76.8)	<0.001
Type 2 diabetes	5 (3.6)	50 (59.5)	3 (3.4)	34 (49.3)	<0.001
Arterial hypertension	41 (29.9)	81 (96.4)	27 (30.7)	67 (97.1)	<0.001
Dyslipidemia	7 (5.1)	41 (48.8)	4 (4.6)	38 (55.1)	<0.001
BMI ≥ 30 kg/m^2^	14 (10.2)	41 (48.8)	16 (18.2)	34 (49.3)	<0.001
Hepatic axis					
CAP, dB/m	210 (188–254)	262 (222–301)	236 (192–274)	297 (252–328)	<0.001
LSM, kPa	5.0 (3.8–6.6)	5.3 (4.1–6.2)	7.2 (5.0–12.8)	8.7 (5.9–12.0)	<0.001
FIB-4 score	1.2 (0.8–1.7)	1.4 (1.1–1.8)	3.6 (2.3–5.8)	3.2 (2.4–5.5)	<0.001
APRI score	0.3 (0.2–0.4)	0.3 (0.2–0.4)	1.0 (0.5–2.0)	0.8 (0.5–1.6)	<0.001
FAST score	0.1 (0.0–0.2)	0.1 (0.0–0.3)	0.4 (0.2–0.5)	0.5 (0.3–0.7)	<0.001
Inflammatory, renal and biochemical markers					
CRP, mg/L	193 (88–267)	191 (79–268)	228 (137–320)	185 (81–304)	0.124
Procalcitonin, ng/mL	0.5 (0.1–3.0)	0.5 (0.2–2.6)	2.2 (0.5–12.4)	2.1 (0.3–23.3)	<0.001
D-dimer, mg/L	1.7 (1.0–4.0)	1.6 (0.9–2.7)	2.5 (1.3–4.2)	2.5 (1.2–4.2)	0.015
Leukocytes, ×10^9^/L	14.0 (10.0–18.7)	11.3 (9.2–16.2)	11.4 (7.5–16.6)	12.9 (8.6–16.2)	0.069
NLR	13.5 (7.5–25.8)	9.0 (5.6–19.9)	12.0 (6.2–30.0)	12.9 (7.2–26.1)	0.227
Platelet count, ×10^9^/L	246 (182–326)	252 (202–292)	138 (103–212)	180 (144–248)	<0.001
SII	2962 (1636–5535)	2301 (1375–4708)	1650 (764–3958)	2531(1449–4750)	0.012
Lactate, mmol/L	2.00 (1.40–2.96)	1.85 (1.10–2.62)	1.93 (1.23–3.07)	2.70 (1.24–3.52)	0.203
eGFR, mL/min/1.73 m^2^	83.6 (60.4–102.2)	69.3 (48.2–90.8)	76.6 (38.0–100.3)	47.4 (27.0–82.2)	<0.001
Glucose, mmol/L	6.7 (5.7–7.6)	8.0 (6.7–10.0)	6.5 (5.9–8.8)	8.2 (6.7–10.1)	<0.001
AST, U/L	24.0 (18.0–34.5)	24.0 (18.0–36.0)	41.0 (23.8–81.8)	52.0 (29.0–88.8)	<0.001
ALT, U/L	23.0 (15.0–41.0)	24.0 (14.0–41.0)	30.5 (19.8–55.5)	38.0 (20.8–73.5)	0.001
GGT, U/L	38 (22–67)	33 (19–71)	45 (24–101)	48 (25–145)	0.028

Continuous variables are presented as median (IQR), and categorical variables as *n* (%). *p*-values were calculated using the Kruskal–Wallis test for continuous variables and χ^2^ or Fisher’s exact test for categorical variables, as appropriate. *p*-values for phenotype-defining variables are shown descriptively only. Admission lactate was available in 227/378 patients (60.1%). CAP, controlled attenuation parameter; LSM, liver stiffness measurement; FIB-4, Fibrosis-4 index; APRI, AST-to-platelet ratio index; FAST, FibroScan-AST score; SOFA, Sequential Organ Failure Assessment; MASLD, metabolic-dysfunction-associated steatotic liver disease; CKD, chronic kidney disease; CRP, C-reactive protein; eGFR, estimated glomerular filtration rate; NLR, neutrophil-to-lymphocyte ratio.

**Table 2:** Clinical outcomes by liver–metabolic phenotype in the whole cohort (*n* = 378).

Outcome	Low Risk (*n* = 137)	CM-Only (*n* = 84)	Liver-Dom. (*n* = 88)	Mixed L + C (*n* = 69)	OR Mixed vs. Low (95% CI)	*p*-Trend
Acute kidney injury (AKI)	19/137 (13.9)	12/84 (14.3)	23/88 (26.1)	28/69 (40.6)	4.24 (2.14–8.39)	<0.001
Continuous renal replacement therapy	7/137 (5.1)	7/84 (8.3)	14/88 (15.9)	18/69 (26.1)	6.55 (2.58–16.63)	<0.001
Septic shock	31/137 (22.6)	15/84 (17.9)	25/88 (28.4)	29/69 (42.0)	2.48 (1.33–4.62)	0.003
ICU admission	40/137 (29.2)	18/84 (21.4)	35/88 (39.8)	33/69 (47.8)	2.22 (1.22–4.05)	0.002
Invasive mechanical ventilation	24/137 (17.5)	12/84 (14.3)	23/88 (26.1)	29/69 (42.0)	3.41 (1.78–6.54)	<0.001
ARDS	24/137 (17.5)	9/84 (10.7)	15/88 (17.0)	24/69 (34.8)	2.51 (1.29–4.87)	0.010
Renal-circulatory composite	45/137 (32.8)	22/84 (26.2)	33/88 (37.5)	37/69 (53.6)	2.36 (1.31–4.27)	0.005
Organ-support composite	47/137 (34.3)	22/84 (26.2)	39/88 (44.3)	36/69 (52.2)	2.09 (1.16–3.77)	0.005

Values are events/*n* (%). Odds ratios compare the Mixed liver–cardiometabolic phenotype with the Low-risk phenotype. The displayed *p*-values represent Cochran–Armitage trend tests across ordered phenotypes.

**Table 3:** Severity-adjusted associations between liver–metabolic phenotypes and organ-support outcomes.

Outcome	CM-Only vs. Low Risk	Liver-Dominant vs. Low Risk	Mixed L + C vs. Low Risk
AKI	1.23 (0.54–2.73); *p* = 0.608	1.62 (0.79–3.3); *p* = 0.184	2.82 (1.37–5.9); *p* = 0.005
CRRT	2.19 (0.72–6.69); *p* = 0.162	2.27 (0.87–6.28); *p* = 0.095	3.87 (1.5–10.8); *p* = 0.005
Septic shock	0.98 (0.47–2); *p* = 0.957	1.02 (0.52–1.96); *p* = 0.964	1.78 (0.89–3.53); *p* = 0.103
ICU admission	0.93 (0.47–1.82); *p* = 0.844	1.27 (0.69–2.34); *p* = 0.441	1.75 (0.89–3.41); *p* = 0.103
Mechanical ventilation	1.07 (0.47–2.33); *p* = 0.875	1.19 (0.58–2.41); *p* = 0.636	2.33 (1.13–4.82); *p* = 0.023
ARDS	0.75 (0.31–1.7); *p* = 0.496	0.68 (0.31–1.44); *p* = 0.317	1.85 (0.89–3.85); *p* = 0.098
Renal–circulatory composite	0.95 (0.5–1.8); *p* = 0.880	0.91 (0.49–1.67); *p* = 0.762	1.71 (0.89–3.3); *p* = 0.109
Organ-support composite	0.92 (0.48–1.75); *p* = 0.807	1.18 (0.65–2.15); *p* = 0.582	1.53 (0.79–2.96); *p* = 0.209

Values are adjusted odds ratios (95% confidence intervals); *p*-values are shown after each estimate. Firth-penalized logistic regression models were adjusted for age, sex and admission SOFA score. The Low-risk phenotype was used as the reference category. Complete case analysis included 371 patients with available covariates. CRRT, continuous renal replacement therapy; ICU, intensive care unit; ARDS, acute respiratory distress syndrome.

## Data Availability

The datasets generated during and/or analyzed during the current study are available from the corresponding author upon reasonable request.

## Supplementary Materials

The following supporting information can be downloaded at: https://www.mdpi.com/article/10.3390/metabo16070468/s1, Table S1. Infection source; Table S2. Full clinical outcomes by liver–metabolic phenotype, including overall χ^2^ *p*-values and *p*-trend; Table S3. Exploratory two-axis logistic models including cardiometabolic burden, liver-risk positivity and their interaction; Table S4. Full severity-adjusted Firth-penalized logistic regression models for organ-support outcomes; Table S5. Sensitivity models for AKI and CRRT using creatinine-based non-renal SOFA adjustment; Table S6. Non-renal SOFA + admission eGFR sensitivity models; Table S7. Sensitivity analyses using individual liver-risk marker definitions; Table S8. Clinical outcomes across liver–metabolic profiles within the MASLD subgroup; Table S9. Severity-adjusted Firth models within the MASLD subgroup (*n* = 170); Table S10. Sequential mortality models; Table S11. Age-adjusted Charlson sensitivity models; Table S12. Benjamini–Hochberg FDR adjustment of phenotype contrasts for the primary renal endpoint; Figure S1. CONSORT-style flow diagram of the SepsisFAT secondary analysis.

## Abbreviations

The following abbreviations are used in this manuscript:

MASLD	Metabolic-dysfunction-associated steatotic liver disease
BMI	Body mass index
APRI	AST to platelet ratio index
AKI	Acute kidney injury
CRRT	Continuous renal replacement therapy
SOFA	Sequential organ failure assessment
eGFR	Estimated glomerular filtration rate
LSM	Liver stiffness measurement
CRP	C-reactive protein
ICU	Intensive care unit
ARDS	Acute respiratory distress syndrome
SRS	Sepsis response signatures
SLD	Steatotic liver disease
CAP	Controlled attenuation parameter
IMV	Invasive mechanical ventilation
NLR	Neutrophil-to-lymphocyte ratio
CCI	Charlson comorbidity index
