# Supplementary material for: Liver–Metabolic Phenotypes and Renal Vulnerability in Community-Acquired Sepsis: Insights from the SepsisFAT Cohort
Source: Metabolites. 2026 Jul 4;16(7):468. doi: 10.3390/metabo16070468 (PMC13414014; doi:10.3390/metabo16070468)
Supplement: Supplementary file 1 [file metabolites-16-00468-s001.zip › metabolites-4386323-supplementary.pdf]

Liver–Metabolic Phenotypes and Renal Vulnerability in Community-Acquired Sepsis: Insights from the SepsisFAT Cohort

Supplementary Table S1. Infection source

Variable	Low risk (n=137)	Cardiometabolic-only (n=84)	Liver-dominant (n=88)	Mixed L+C (n=69)	p-value
**Infection source, n (%)**					
Respiratory	49 (35.8)	19 (22.6)	26 (29.5)	24 (34.8)	0.261
Urinary	29 (21.2)	12 (14.3)	19 (21.6)	11 (15.9)	
Intra-abdominal/GI	20 (14.6)	18 (21.4)	7 (8.0)	10 (14.5)	
Skin and soft tissue	12 (8.8)	14 (16.7)	14 (15.9)	13 (18.8)	
Central nervous system	14 (10.2)	9 (10.7)	10 (11.4)	4 (5.8)	
Other / unknown	13 (9.5)	12 (14.3)	12 (13.6)	7 (10.1)	
**Positive blood culture (bacteremia)**	36 (26.3)	19 (22.6)	19 (21.6)	25 (36.2)	0.163

Supplementary Table S2. Full clinical outcomes by liver–metabolic phenotype, including overall χ^2 p-values and p-trend

Outcome	Low risk (n=137)	CM-only (n=84)	Liver-dom. (n=88)	Mixed L+C (n=69)	OR Mixed vs Low (95% CI)	p-value (χ^2)	p-trend
Acute kidney injury (AKI)	19/137 (13.9)	12/84 (14.3)	23/88 (26.1)	28/69 (40.6)	4.24 (2.14–8.39)	<0.001	<0.001
Continuous renal replacement therapy (CRRT)	7/137 (5.1)	7/84 (8.3)	14/88 (15.9)	18/69 (26.1)	6.55 (2.58–16.63)	<0.001	<0.001
Septic shock	31/137 (22.6)	15/84 (17.9)	25/88 (28.4)	29/69 (42.0)	2.48 (1.33–4.62)	0.005	0.003
ICU admission	40/137 (29.2)	18/84 (21.4)	35/88 (39.8)	33/69 (47.8)	2.22 (1.22–4.05)	0.002	0.002
Invasive mechanical ventilation	24/137 (17.5)	12/84 (14.3)	23/88 (26.1)	29/69 (42.0)	3.41 (1.78–6.54)	<0.001	<0.001
ARDS	24/137 (17.5)	9/84 (10.7)	15/88 (17.0)	24/69 (34.8)	2.51 (1.29–4.87)	0.002	0.010
Renal-circulatory composite (AKI / CRRT / shock)	45/137 (32.8)	22/84 (26.2)	33/88 (37.5)	37/69 (53.6)	2.36 (1.31–4.27)	0.004	0.005
Organ-support composite (ICU / IMV / CRRT / shock)	47/137 (34.3)	22/84 (26.2)	39/88 (44.3)	36/69 (52.2)	2.09 (1.16–3.77)	0.004	0.005

Supplementary Table S3. Exploratory two-axis logistic models including cardiometabolic burden, liver-risk positivity and their interaction

Outcome	Term	OR_95CI	p_value
AKI	Intercept	0.24 (0.14–0.38)	<0.001
AKI	Cardiometabolic burden	1.08 (0.47–2.39)	0.861
AKI	Liver-risk positivity	2.31 (1.13–4.75)	0.022
AKI	Interaction: CM burden × liver-risk	1.40 (0.48–4.16)	0.541
CRRT	Intercept	0.06 (0.02–0.11)	<0.001
CRRT	Cardiometabolic burden	1.72 (0.57–5.20)	0.330
CRRT	Liver-risk positivity	3.48 (1.38–9.55)	0.010
CRRT	Interaction: CM burden × liver-risk	1.07 (0.28–4.16)	0.919
Septic shock	Intercept	0.30 (0.20–0.44)	<0.001
Septic shock	Cardiometabolic burden	0.76 (0.37–1.50)	0.441
Septic shock	Liver-risk positivity	1.35 (0.73–2.50)	0.334
Septic shock	Interaction: CM burden × liver-risk	2.42 (0.93–6.40)	0.071
ICU admission	Intercept	0.42 (0.29–0.60)	<0.001
ICU admission	Cardiometabolic burden	0.68 (0.35–1.27)	0.236
ICU admission	Liver-risk positivity	1.57 (0.89–2.76)	0.118
ICU admission	Interaction: CM burden × liver-risk	2.05 (0.83–5.10)	0.121
Invasive mechanical ventilation	Intercept	0.22 (0.14–0.33)	<0.001
Invasive mechanical ventilation	Cardiometabolic burden	0.79 (0.36–1.66)	0.547
Invasive mechanical ventilation	Liver-risk positivity	1.64 (0.85–3.14)	0.137
Invasive mechanical ventilation	Interaction: CM burden × liver-risk	2.58 (0.95–7.24)	0.066
ARDS	Intercept	0.30 (0.19–0.47)	<0.001
ARDS	Cardiometabolic burden	0.58 (0.24–1.30)	0.200
ARDS	Liver-risk positivity	0.98 (0.46–2.03)	0.958
ARDS	Interaction: CM burden × liver-risk	3.54 (1.16–11.38)	0.030

Renal-circulatory composite	Intercept	0.69 (0.47–1.01)	0.058
Renal-circulatory composite	Cardiometabolic burden	0.74 (0.39–1.39)	0.354
Renal-circulatory composite	Liver-risk positivity	1.29 (0.70–2.36)	0.411
Renal-circulatory composite	Interaction: CM burden × liver-risk	1.94 (0.77–4.94)	0.164
Organ-support composite	Intercept	0.55 (0.38–0.78)	<0.001
Organ-support composite	Cardiometabolic burden	0.68 (0.37–1.24)	0.216
Organ-support composite	Liver-risk positivity	1.49 (0.86–2.59)	0.159
Organ-support composite	Interaction: CM burden × liver-risk	1.97 (0.82–4.76)	0.130
In-hospital mortality	Intercept	0.11 (0.06–0.19)	<0.001
In-hospital mortality	Cardiometabolic burden	0.92 (0.35–2.26)	0.867
In-hospital mortality	Liver-risk positivity	1.39 (0.60–3.16)	0.435
In-hospital mortality	Interaction: CM burden × liver-risk	2.42 (0.72–8.47)	0.157

Supplementary Table S4. Full severity-adjusted Firth-penalized logistic regression models for organ-support outcomes (n=371)

Outcome	Events	Variable	beta	SE	aOR	95% CI	p-value
AKI	81	Age, per year	0.002	0.008	1	0.99–1.02	0.834
AKI	81	Male sex	0.351	0.27	1.42	0.84–2.43	0.195
AKI	81	SOFA score	0.231	0.048	1.26	1.15–1.39	<0.001
AKI	81	Cardiometabolic-only vs Low risk	0.21	0.406	1.23	0.54–2.73	0.608
AKI	81	Liver-dominant vs Low risk	0.481	0.36	1.62	0.79–3.3	0.184
AKI	81	Mixed L+C vs Low risk	1.038	0.37	2.82	1.37–5.9	0.005
CRRT	45	Age, per year	-0.001	0.011	1	0.98–1.02	0.929
CRRT	45	Male sex	0.478	0.344	1.61	0.82–3.25	0.169
CRRT	45	SOFA score	0.273	0.056	1.31	1.18–1.47	<0.001
CRRT	45	Cardiometabolic-only vs Low risk	0.786	0.551	2.19	0.72–6.69	0.162
CRRT	45	Liver-dominant vs Low risk	0.82	0.491	2.27	0.87–6.28	0.095
CRRT	45	Mixed L+C vs Low risk	1.354	0.492	3.87	1.5–10.8	0.005
Septic shock	98	Age, per year	-0.006	0.008	0.99	0.98–1.01	0.446
Septic shock	98	Male sex	0.199	0.252	1.22	0.74–2.01	0.432
Septic shock	98	SOFA score	0.261	0.047	1.3	1.19–1.43	<0.001
Septic shock	98	Cardiometabolic-only vs Low risk	-0.02	0.366	0.98	0.47–2	0.957
Septic shock	98	Liver-dominant vs Low risk	0.015	0.335	1.02	0.52–1.96	0.964
Septic shock	98	Mixed L+C vs Low risk	0.574	0.35	1.78	0.89–3.53	0.103
ICU admission	124	Age, per year	-0.016	0.007	0.98	0.97–1	0.031
ICU admission	124	Male sex	0.072	0.238	1.07	0.67–1.72	0.764
ICU admission	124	SOFA score	0.28	0.048	1.32	1.21–1.46	<0.001
ICU admission	124	Cardiometabolic-only vs Low risk	-0.068	0.344	0.93	0.47–1.82	0.844
ICU admission	124	Liver-dominant vs Low risk	0.241	0.311	1.27	0.69–2.34	0.441

ICU admission	124	Mixed L+C vs Low risk	0.557	0.34	1.75	0.89–3.41	0.103
Invasive mechanical ventilation	86	Age, per year	-0.002	0.008	1	0.98–1.01	0.793
Invasive mechanical ventilation	86	Male sex	0.364	0.271	1.44	0.84–2.47	0.181
Invasive mechanical ventilation	86	SOFA score	0.303	0.05	1.35	1.23–1.5	<0.001
Invasive mechanical ventilation	86	Cardiometabolic-only vs Low risk	0.064	0.402	1.07	0.47–2.33	0.875
Invasive mechanical ventilation	86	Liver-dominant vs Low risk	0.172	0.36	1.19	0.58–2.41	0.636
Invasive mechanical ventilation	86	Mixed L+C vs Low risk	0.844	0.368	2.33	1.13–4.82	0.023
ARDS	71	Age, per year	-0.003	0.009	1	0.98–1.01	0.700
ARDS	71	Male sex	0.317	0.282	1.37	0.79–2.41	0.264
ARDS	71	SOFA score	0.254	0.049	1.29	1.17–1.43	<0.001
ARDS	71	Cardiometabolic-only vs Low risk	-0.29	0.427	0.75	0.31–1.7	0.496
ARDS	71	Liver-dominant vs Low risk	-0.387	0.389	0.68	0.31–1.44	0.317
ARDS	71	Mixed L+C vs Low risk	0.618	0.37	1.85	0.89–3.85	0.098
Renal-circulatory composite	135	Age, per year	-0.007	0.007	0.99	0.98–1.01	0.311
Renal-circulatory composite	135	Male sex	0.247	0.233	1.28	0.81–2.03	0.289
Renal-circulatory composite	135	SOFA score	0.287	0.048	1.33	1.22–1.47	<0.001
Renal-circulatory composite	135	Cardiometabolic-only vs Low risk	-0.049	0.326	0.95	0.5–1.8	0.880
Renal-circulatory composite	135	Liver-dominant vs Low risk	-0.094	0.311	0.91	0.49–1.67	0.762

Renal-circulatory composite	135	Mixed L+C vs Low risk	0.536	0.333	1.71	0.89–3.3	0.109
Organ-support composite	142	Age, per year	-0.011	0.007	0.99	0.97–1	0.105
Organ-support composite	142	Male sex	0.143	0.232	1.15	0.73–1.82	0.540
Organ-support composite	142	SOFA score	0.308	0.05	1.36	1.24–1.51	<0.001
Organ-support composite	142	Cardiometabolic-only vs Low risk	-0.08	0.326	0.92	0.48–1.75	0.807
Organ-support composite	142	Liver-dominant vs Low risk	0.168	0.305	1.18	0.65–2.15	0.582
Organ-support composite	142	Mixed L+C vs Low risk	0.423	0.336	1.53	0.79–2.96	0.209

Supplementary Table S5. Sensitivity models for AKI and CRRT using creatinine-based non-renal SOFA adjustment (n=369)

Outcome	Events	Variable	beta	SE	aOR	95% CI	p-value
AKI	81	Age, per year	0.003	0.008	1	0.99–1.02	0.678
AKI	81	Male sex	0.391	0.268	1.48	0.87–2.52	0.146
AKI	81	Non-renal SOFA score	0.226	0.053	1.25	1.13–1.39	<0.001
AKI	81	Cardiometabolic-only vs Low risk	0.214	0.405	1.24	0.55–2.73	0.601
AKI	81	Liver-dominant vs Low risk	0.559	0.355	1.75	0.87–3.54	0.117
AKI	81	Mixed L+C vs Low risk	1.152	0.364	3.16	1.55–6.55	0.002
CRRT	45	Age, per year	0.001	0.011	1	0.98–1.02	0.919
CRRT	45	Male sex	0.517	0.341	1.68	0.86–3.36	0.133
CRRT	45	Non-renal SOFA score	0.277	0.062	1.32	1.17–1.50	<0.001
CRRT	45	Cardiometabolic-only vs Low risk	0.791	0.549	2.21	0.73–6.70	0.158
CRRT	45	Liver-dominant vs Low risk	0.921	0.484	2.51	0.98–6.88	0.055
CRRT	45	Mixed L+C vs Low risk	1.49	0.486	4.44	1.74–12.22	0.002

Supplementary Table S6. Non-renal SOFA + admission eGFR sensitivity models

Outcome	n	Events	Variable	beta	SE	aOR	95% CI	p-value
AKI	369	81	Age, per year	-0.01	0.01	1.00	0.98–1.01	0.605
AKI	369	81	Male sex	0.34	0.27	1.40	0.82–2.41	0.213
AKI	369	81	Non-renal SOFA score	0.21	0.05	1.23	1.11–1.37	<0.001
AKI	369	81	Admission eGFR, per mL/min/1.73 m²	-0.01	0.00	0.99	0.98–1.00	0.036
AKI	369	81	Cardiometabolic-only vs Low risk	0.16	0.41	1.17	0.52–2.60	0.696
AKI	369	81	Liver-dominant vs Low risk	0.49	0.36	1.63	0.80–3.32	0.178
AKI	369	81	Mixed L+C vs Low risk	1.00	0.37	2.72	1.31–5.71	0.007
CRRT	369	45	Age, per year	-0.01	0.01	0.99	0.97–1.02	0.509
CRRT	369	45	Male sex	0.50	0.34	1.64	0.83–3.32	0.153
CRRT	369	45	Non-renal SOFA score	0.26	0.06	1.29	1.14–1.47	<0.001
CRRT	369	45	Admission eGFR, per mL/min/1.73 m²	-0.01	0.01	0.99	0.98–1.00	0.049
CRRT	369	45	Cardiometabolic-only vs Low risk	0.75	0.55	2.12	0.70–6.46	0.182
CRRT	369	45	Liver-dominant vs Low risk	0.83	0.49	2.29	0.88–6.33	0.091
CRRT	369	45	Mixed L+C vs Low risk	1.32	0.49	3.73	1.43–10.43	0.007

Supplementary Table S7. Sensitivity analyses using individual liver-risk marker definitions

Among the 157 liver-risk-positive patients, 122 (77.7%) fulfilled at least one blood-based criterion (FIB-4 or APRI), 80 (51.0%) fulfilled at least one elastography-based criterion (LSM or FAST), and 45 (28.7%) fulfilled both, indicating overlapping but non-identical routes to liver-risk classification.

Mixed liver–cardiometabolic phenotype adjusted odds ratios (vs Low risk reference) for renal outcomes and in-hospital mortality, by liver-risk marker definition. All aOR estimates from Firth-penalized logistic regression adjusted for age, sex and admission SOFA score. n = 378 total cohort.

**Liver-risk definition**	**AKI aOR (95% CI)**	**CRRT aOR (95% CI)**	**Mortality aOR (95% CI)**
Any of 4 markers (primary analysis)	2.82 (1.37–5.90) *	3.87 (1.50–10.80) *	1.78 (0.76–4.15)
FIB-4 ≥ 2.67 only	4.98 (2.23–11.12) *	5.79 (2.12–15.82) *	2.65 (1.09–6.45) *
APRI ≥ 1.0 only	5.31 (2.11–13.39) *	8.49 (2.95–24.41) *	2.98 (1.08–8.19) *
LSM ≥ 10 kPa only	1.20 (0.42–3.44)	0.92 (0.23–3.72)	1.51 (0.48–4.77)
FAST ≥ 0.55 only	2.91 (1.15–7.38) *	3.90 (1.36–11.14) *	2.32 (0.80–6.71)

Supplementary Table S8. Clinical outcomes across liver–metabolic profiles within the MASLD subgroup

Outcome	Low-risk MASLD (n=35)	Cardiometabolic-predominant MASLD (n=50)	Liver-specific /fibroinflammatory MASLD (n=36)	Mixed liver–cardiometabolic MASLD (n=53)	OR Mixed vs Low (95% CI)	p-value (χ^2)	p-trend
Acute kidney injury (AKI)	4/35 (11.4)	9/50 (18.0)	10/36 (27.8)	23/53 (43.4)	5.94 (1.84–19.23)	0.003	<0.001
Continuous renal replacement therapy (CRRT)	2/35 (5.7)	5/50 (10.0)	6/36 (16.7)	15/53 (28.3)	6.51 (1.39–30.61)	0.018	0.002
Septic shock	11/35 (31.4)	9/50 (18.0)	10/36 (27.8)	23/53 (43.4)	1.67 (0.68–4.10)	0.046	0.067
ICU admission	10/35 (28.6)	11/50 (22.0)	16/36 (44.4)	27/53 (50.9)	2.60 (1.05–6.45)	0.011	0.004
Invasive mechanical ventilation	8/35 (22.9)	7/50 (14.0)	13/36 (36.1)	23/53 (43.4)	2.59 (0.99–6.74)	0.007	0.003
ARDS	8/35 (22.9)	6/50 (12.0)	7/36 (19.4)	19/53 (35.8)	1.89 (0.72–4.97)	0.035	0.045
Renal-circulatory composite (AKI / CRRT / shock)	13/35 (37.1)	14/50 (28.0)	13/36 (36.1)	30/53 (56.6)	2.21 (0.92–5.29)	0.024	0.019
Organ-support composite (ICU / IMV / CRRT / shock)	14/35 (40.0)	14/50 (28.0)	19/36 (52.8)	29/53 (54.7)	1.81 (0.76–4.31)	0.029	0.026
In-hospital mortality	5/35 (14.3)	6/50 (12.0)	5/36 (13.9)	16/53 (30.2)	2.59 (0.85–7.90)	0.067	0.031

Supplementary Table S9. Severity-adjusted Firth models within the MASLD subgroup (n=170)

Outcome	Events	Variable	beta	SE	aOR	95% CI	p-value
AKI	46	Age, per year	0.007	0.016	1.01	0.98–1.04	0.650
AKI	46	Male sex	1.029	0.448	2.8	1.17–7.13	0.021
AKI	46	SOFA score	0.514	0.093	1.67	1.41–2.05	<0.001
AKI	46	Cardiometabolic-predominant MASLD vs Low-risk MASLD	1.415	0.714	4.12	1.05–19.13	0.042
AKI	46	Liver-specific/fibroinflammatory MASLD vs Low-risk MASLD	0.558	0.738	1.75	0.40–8.21	0.458
AKI	46	Mixed liver–cardiometabolic MASLD vs Low-risk MASLD	1.444	0.653	4.24	1.22–17.39	0.022
CRRT	28	Age, per year	0.012	0.019	1.01	0.97–1.05	0.563
CRRT	28	Male sex	0.443	0.52	1.56	0.55–4.69	0.410
CRRT	28	SOFA score	0.534	0.103	1.71	1.41–2.15	<0.001
CRRT	28	Cardiometabolic-predominant MASLD vs Low-risk MASLD	1.385	0.876	3.99	0.74–28.52	0.109
CRRT	28	Liver-specific/fibroinflammatory MASLD vs Low-risk MASLD	0.281	0.899	1.32	0.22–9.42	0.762
CRRT	28	Mixed liver–cardiometabolic MASLD vs Low-risk MASLD	1.218	0.785	3.38	0.77–20.45	0.110
Septic shock	52	Age, per year	0.01	0.014	1.01	0.98–1.04	0.492
Septic shock	52	Male sex	0.047	0.381	1.05	0.49–2.24	0.904
Septic shock	52	SOFA score	0.382	0.078	1.47	1.27–1.73	<0.001
Septic shock	52	Cardiometabolic-predominant MASLD vs Low-risk MASLD	-0.282	0.556	0.75	0.25–2.28	0.616
Septic shock	52	Liver-specific/fibroinflammatory MASLD vs Low-risk MASLD	-0.545	0.591	0.58	0.17–1.85	0.358
Septic shock	52	Mixed liver–cardiometabolic MASLD vs Low-risk MASLD	0.133	0.511	1.14	0.42–3.18	0.797

ICU admission	63	Age, per year	-0.038	0.017	0.96	0.93–1.00	0.029
ICU admission	63	Male sex	-0.625	0.438	0.54	0.22–1.27	0.157
ICU admission	63	SOFA score	0.812	0.136	2.25	1.76–3.07	<0.001
ICU admission	63	Cardiometabolic-predominant MASLD vs Low-risk MASLD	0.782	0.644	2.19	0.62–8.42	0.225
ICU admission	63	Liver-specific/fibroinflammatory MASLD vs Low-risk MASLD	0.909	0.663	2.48	0.67–9.83	0.174
ICU admission	63	Mixed liver–cardiometabolic MASLD vs Low-risk MASLD	0.987	0.618	2.68	0.81–9.82	0.109
Invasive mechanical ventilation	50	Age, per year	-0.023	0.017	0.98	0.94–1.01	0.189
Invasive mechanical ventilation	50	Male sex	0.002	0.459	1	0.39–2.54	0.997
Invasive mechanical ventilation	50	SOFA score	0.774	0.128	2.17	1.71–2.89	<0.001
Invasive mechanical ventilation	50	Cardiometabolic-predominant MASLD vs Low-risk MASLD	0.615	0.718	1.85	0.45–8.48	0.398
Invasive mechanical ventilation	50	Liver-specific/fibroinflammatory MASLD vs Low-risk MASLD	0.703	0.718	2.02	0.48–9.12	0.337
Invasive mechanical ventilation	50	Mixed liver–cardiometabolic MASLD vs Low-risk MASLD	0.847	0.66	2.33	0.64–9.55	0.202
ARDS	39	Age, per year	-0.023	0.016	0.98	0.94–1.01	0.158
ARDS	39	Male sex	-0.288	0.448	0.75	0.30–1.84	0.530
ARDS	39	SOFA score	0.477	0.089	1.61	1.36–1.95	<0.001
ARDS	39	Cardiometabolic-predominant MASLD vs Low-risk MASLD	0.074	0.654	1.08	0.29–4.09	0.912
ARDS	39	Liver-specific/fibroinflammatory MASLD vs Low-risk MASLD	-0.716	0.722	0.49	0.11–2.03	0.327
ARDS	39	Mixed liver–cardiometabolic MASLD vs Low-risk MASLD	0.403	0.582	1.5	0.48–4.97	0.493

Renal-circulatory composite	69	Age, per year	0.002	0.014	1	0.98–1.03	0.861
Renal-circulatory composite	69	Male sex	0.555	0.375	1.74	0.83–3.70	0.141
Renal-circulatory composite	69	SOFA score	0.491	0.09	1.63	1.38–1.99	<0.001
Renal-circulatory composite	69	Cardiometabolic-predominant MASLD vs Low-risk MASLD	0.237	0.531	1.27	0.45–3.71	0.657
Renal-circulatory composite	69	Liver-specific/fibroinflammatory MASLD vs Low-risk MASLD	-0.457	0.59	0.63	0.19–2.03	0.443
Renal-circulatory composite	69	Mixed liver–cardiometabolic MASLD vs Low-risk MASLD	0.469	0.513	1.6	0.58–4.49	0.364
Organ-support composite	75	Age, per year	-0.016	0.016	0.98	0.95–1.02	0.312
Organ-support composite	75	Male sex	-0.48	0.399	0.62	0.28–1.36	0.232
Organ-support composite	75	SOFA score	0.725	0.123	2.06	1.65–2.72	<0.001
Organ-support composite	75	Cardiometabolic-predominant MASLD vs Low-risk MASLD	0.195	0.564	1.22	0.40–3.81	0.732
Organ-support composite	75	Liver-specific/fibroinflammatory MASLD vs Low-risk MASLD	0.56	0.597	1.75	0.54–5.87	0.354
Organ-support composite	75	Mixed liver–cardiometabolic MASLD vs Low-risk MASLD	0.3	0.553	1.35	0.45–4.13	0.592
In-hospital mortality	32	Age, per year	0.042	0.019	1.04	1.01–1.09	0.023
In-hospital mortality	32	Male sex	0.29	0.452	1.34	0.54–3.38	0.530
In-hospital mortality	32	SOFA score	0.424	0.091	1.53	1.29–1.87	<0.001
In-hospital mortality	32	Cardiometabolic-predominant MASLD vs Low-risk MASLD	0.096	0.681	1.1	0.28–4.50	0.890

In-hospital mortality	32	Liver-specific/fibroinflammatory MASLD vs Low-risk MASLD	-0.761	0.75	0.47	0.10–2.09	0.318
In-hospital mortality	32	Mixed liver–cardiometabolic MASLD vs Low-risk MASLD	0.196	0.617	1.22	0.36–4.41	0.754

Supplementary Table S10. Sequential mortality models

Model	n	deaths	OR	OR (95% CI)	p-value
Unadjusted	378	52	3.1	3.10 (1.43–6.70)	0.004
Age + sex	375	51	2.86	2.86 (1.29–6.38)	0.010
Age + sex + respiratory source	375	51	3.01	3.01 (1.33–6.81)	0.008
Age + sex + respiratory source + SOFA	371	51	1.92	1.92 (0.80–4.58)	0.142

Supplementary Table S11. Age-adjusted Charlson sensitivity models

Model	n	deaths	OR	OR (95% CI)	p-value
Sex + age-adjusted CCI	375	52	2.82	2.82 (1.28–6.20)	0.010
Sex + age-adjusted CCI + respiratory source	375	52	2.99	2.99 (1.33–6.72)	0.008
Sex + age-adjusted CCI + respiratory source + SOFA	371	52	2.04	2.04 (0.86–4.82)	0.104

Supplementary Table S12. Benjamini–Hochberg FDR adjustment of phenotype contrasts for the primary endpoint (AKI) and the key secondary endpoint (CRRT).

Panel A. Crude (unadjusted) Firth-penalized models (n = 378)

Outcome	Contrast	OR (95% CI)	Raw p	BH-adjusted q
AKI	Cardiometabolic-only vs Low	1.05 (0.48–2.27)	0.906	0.906
AKI	Liver-dominant vs Low	2.18 (1.11–4.28)	0.024	0.035
AKI	Mixed L+C vs Low	4.17 (2.12–8.23)	<0.001	<0.001
CRRT	Cardiometabolic-only vs Low	1.68 (0.59–4.84)	0.333	0.400
CRRT	Liver-dominant vs Low	3.39 (1.34–8.59)	0.010	0.020
CRRT	Mixed L+C vs Low	6.25 (2.51–15.56)	<0.001	<0.001

Panel B. Severity-adjusted Firth-penalized models (age + sex + admission SOFA, n = 371)

Outcome	Contrast	aOR (95% CI)	Raw p	BH-adjusted q
AKI	Cardiometabolic-only vs Low	1.23 (0.55–2.76)	0.608	0.608
AKI	Liver-dominant vs Low	1.62 (0.79–3.30)	0.186	0.223
AKI	Mixed L+C vs Low	2.82 (1.36–5.88)	0.006	0.020
CRRT	Cardiometabolic-only vs Low	2.19 (0.74–6.54)	0.158	0.223
CRRT	Liver-dominant vs Low	2.27 (0.86–6.01)	0.099	0.198
CRRT	Mixed L+C vs Low	3.87 (1.46–10.29)	0.007	0.020

Benjamini–Hochberg adjustment was applied separately within each analysis layer (six tests per layer: two outcomes × three phenotype contrasts versus the Low-risk reference). AKI, acute kidney injury; CRRT, continuous renal replacement therapy; OR, odds ratio; aOR, adjusted odds ratio; CI, confidence interval; SOFA, Sequential Organ Failure Assessment.

Figure S1. CONSORT style flow diagram

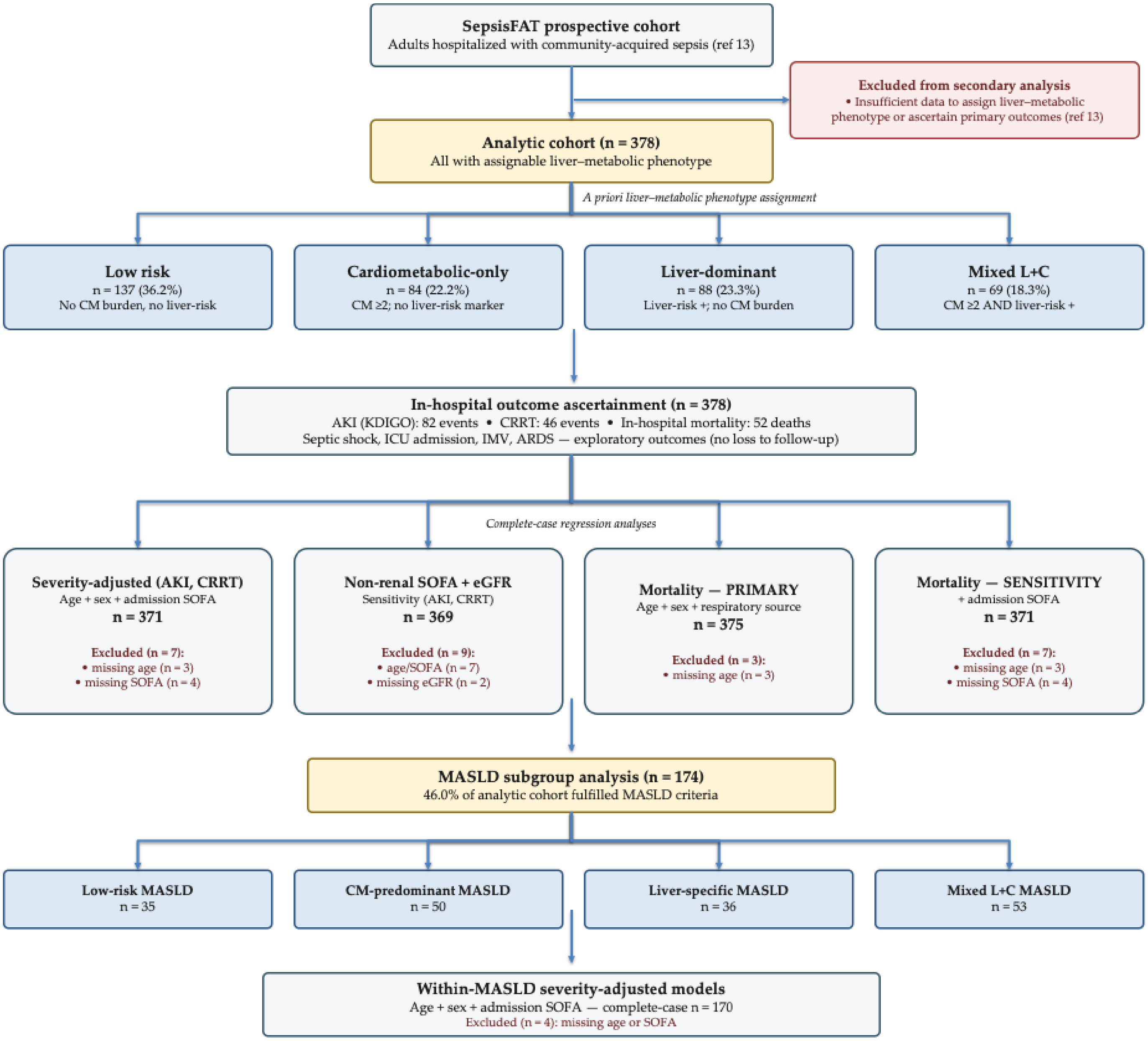

Missing data handling

Phenotype assignment was possible for all n = 378 patients in the analytic cohort. No imputation was performed; all regression analyses were complete-case. Phenotype was assignable when at least one of FIB-4, APRI, FAST or LSM was available (positivity defined by ≥1 marker reaching threshold). Outcome events not documented in the medical record were treated as "no event" (AKI: 93 patients without documented AKI status were treated as AKI-negative; CRRT: 7; in-hospital mortality complete). Within-MASLD models additionally required complete data for age, sex and admission SOFA.
